# Acute respiratory distress syndrome caused by carbon monoxide poisoning and inhalation injury recovered after extracorporeal membrane oxygenation along with direct hemoperfusion with polymyxin B-immobilized fiber column: a case report

**DOI:** 10.1186/s13256-021-03023-w

**Published:** 2021-09-15

**Authors:** Ji Hoon Jang, Hang Jea Jang, Hyun-Kuk Kim, Jin Han Park, Hyo-Jung Kim, Kyeong Min Jo, Woon Heo, Se Hun Kim, Tae-Hoon No, Jae Ha Lee

**Affiliations:** 1 Department of Internal Medicine, Haeundae Paik Hospital, Inje University College of Medicine, Busan, 135-710 Korea; 2 Department of Thoracic and Cardiovascular surgery, Haeundae Paik Hospital, Inje University College of Medicine, Busan, Korea; 3 Department of Anesthesiology and Pain medicine, Haeundae Paik Hospital, Inje University College of Medicine, Busan, Korea; 4 Department of Internal Medicine, Seoul ST. Mary’s Hospital, The Catholic University of Korea, Seoul, Korea

**Keywords:** Carbon monoxide poisoning, Smoke inhalation injury, Acute respiratory distress syndrome

## Abstract

**Background:**

Inhalation injury from smoke or chemical products and carbon monoxide poisoning are major causes of death in burn patients from fire accidents. Respiratory tract injuries from inhalation injury and carbon monoxide poisoning can lead to acute respiratory distress syndrome and cytokine storm syndrome. In the case of acute respiratory failure needing mechanical ventilation accompanied by cytokine storm, mortality is high and immediate adequate treatment at the emergency department is very important.

**Case presentation:**

This report describes a case of acute respiratory distress syndrome and cytokine storm followed by carbon monoxide poisoning in a 34-year-old Korean male patient who was in a house fire, and was successfully treated by extracorporeal membrane oxygenation and direct hemoperfusion with polymyxin B-immobilized fiber column at emergency department.

**Conclusions:**

To prevent mortality in acute respiratory distress syndrome with cytokine storm from inhalation injury and to promote a better prognosis, we suggest that early implication of extracorporeal membranous oxygenation along with direct hemoperfusion with polymyxin B-immobilized fiber column even at the emergency department should be considered.

## Background

Inhalation injury is considered an important cause of morbidity and mortality in patients with burns from fire accidents. Direct thermal injury in the upper airway, smoke-induced chemical irritation in the lower airway, and systematic chemical or metabolic injury with various chemicals constitute the major pathophysiological background that can lead to severe acute respiratory distress syndrome (ARDS) [1]. Carbon monoxide (CO) poisoning is a leading cause of death among poisoning deaths worldwide and is most often caused by house fires. In the case of toxic concentration, increases in carboxyhemoglobin (COHb) interfere with gas exchange in respiratory system, resulting in hypoxemia [2]. Hypoxemia from CO poisoning eventually causes tissue damage, which triggers an inflammatory response [3]. In terms of severe ARDS after inhalation injury, mortality is very high and aggressive treatment is needed in addition to conventional therapy including fluid restriction, protective ventilation, prone positioning, or nitric oxide inhalation [4]. Systemic inflammation caused by a “cytokine storm” due to excessive circulating cytokines also requires attention. Here, we present a case of CO intoxication and inhalation injury with secondary ARDS and cytokine storm in a patient who recovered after treatment with extracorporeal membrane oxygenation (ECMO) and direct hemoperfusion with polymyxin B-immobilized fiber (PMX-DHP) at the emergency department (ED).

## Case presentation

**Fig. 1:**
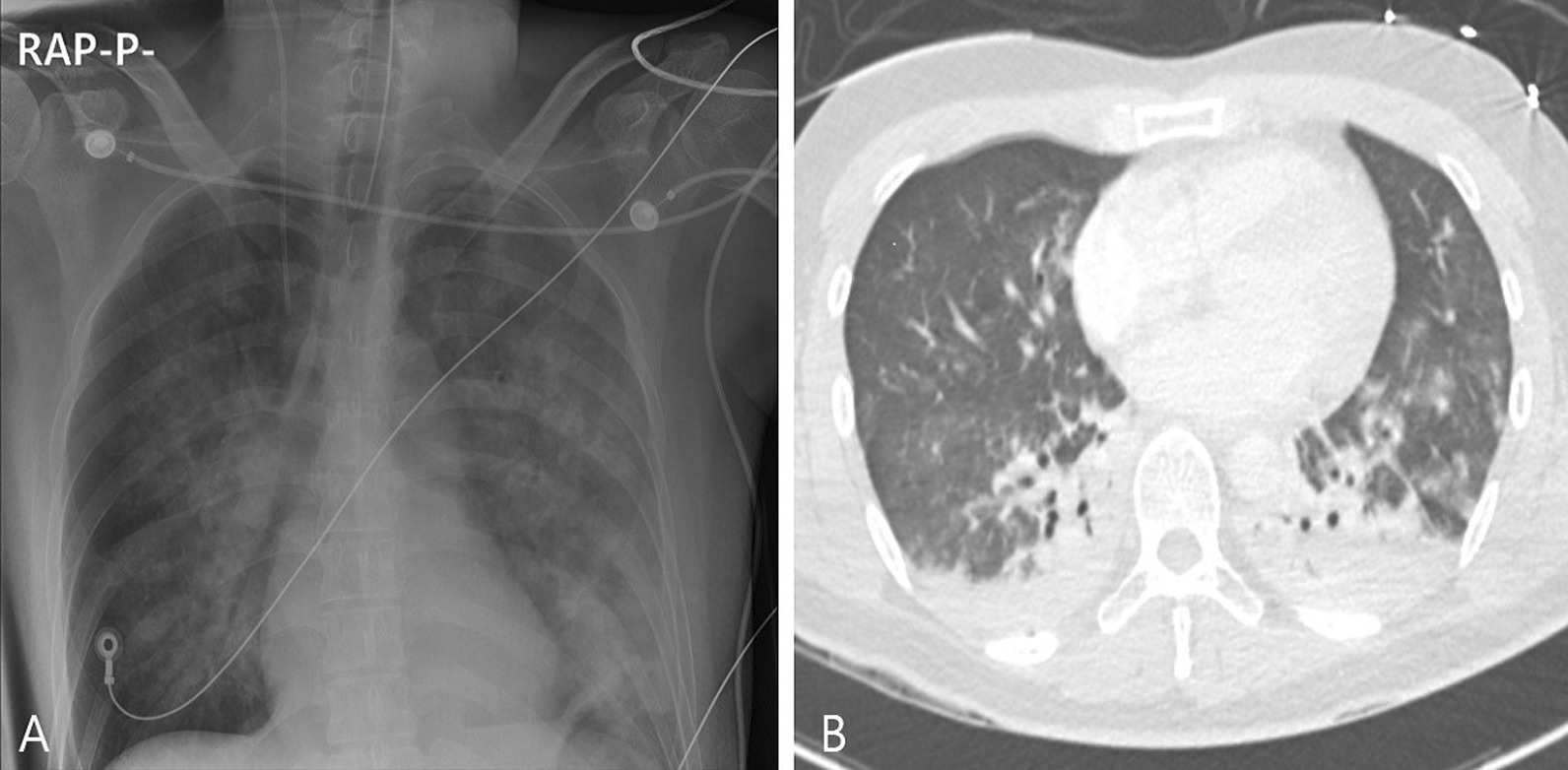
Chest imaging findings. **A** Bilateral infiltrates on chest X-ray. **B** Anterior–posterior density gradient by consolidation in bilateral dependent area with background diffuse ground-glass opacities

**Fig. 2:**
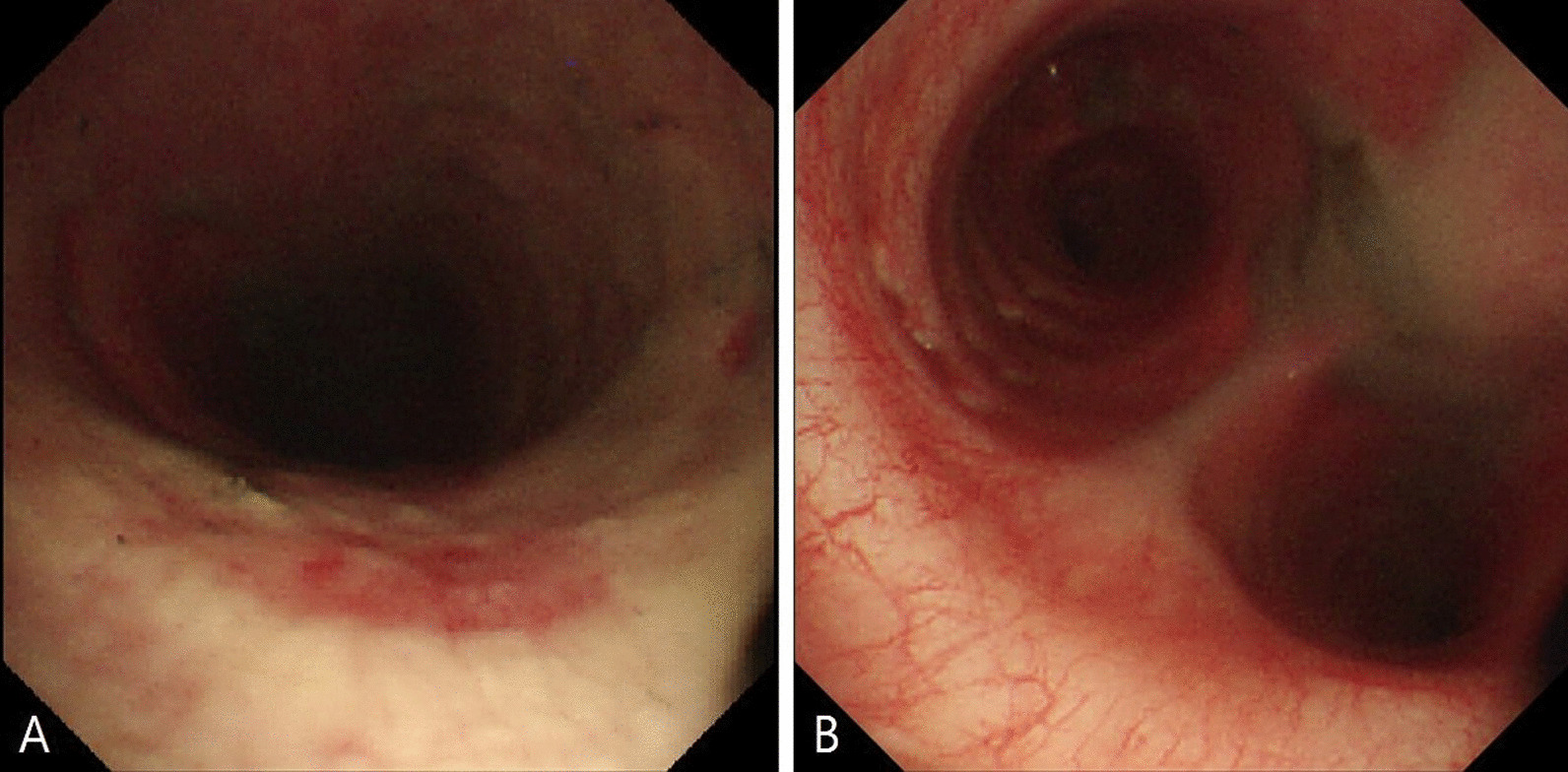
Bronchoscopic findings. **A** Carbon soot disposition with edematous mucosal wall in upper trachea. **B** Hyperemic change with edema and carbon soot disposition in mucosal wall of carina and both main bronchi

**Fig. 3:**
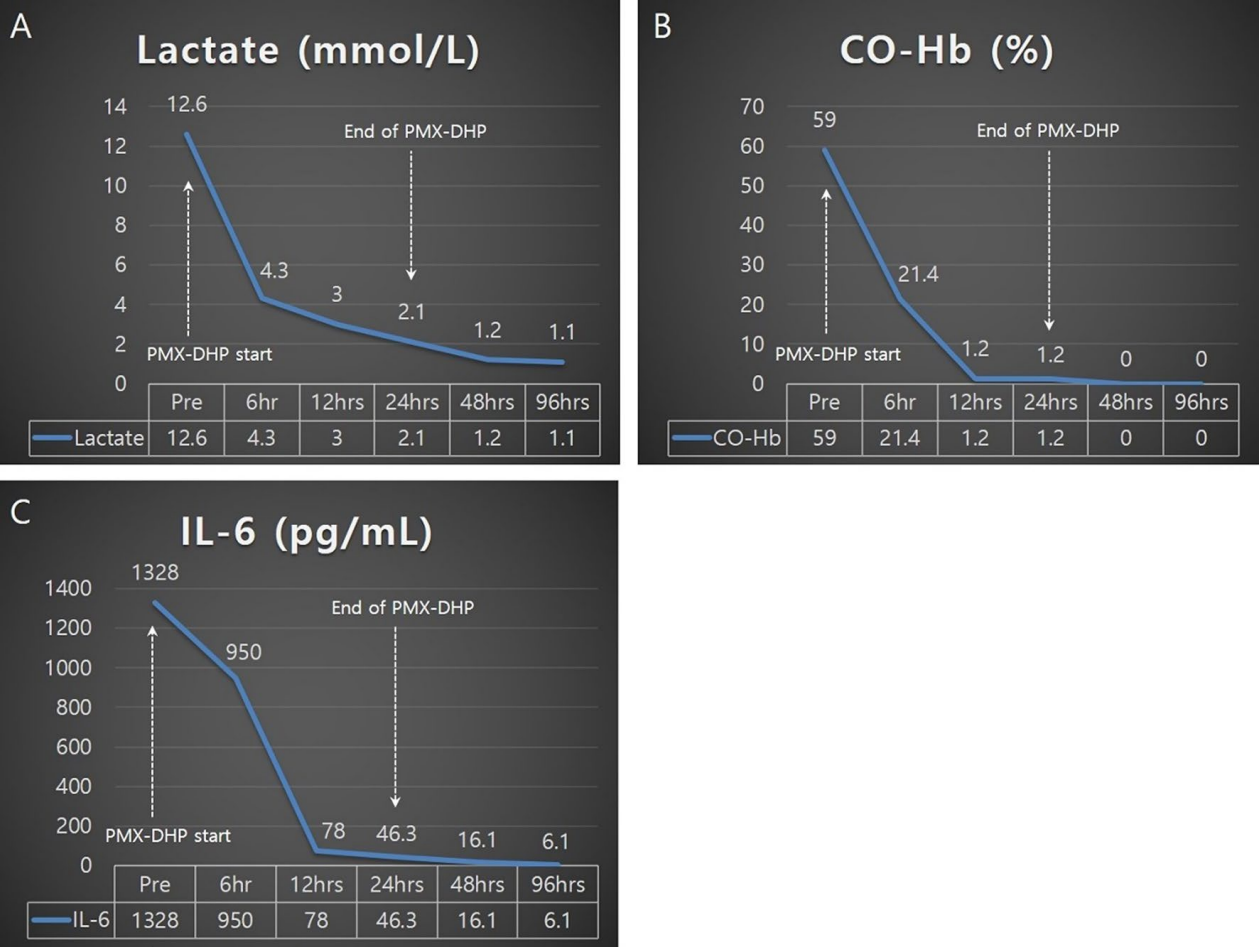
Comparison of serum level of lactate (**A**), carboxyhemoglobin (CO-Hb) (**B**), and interleukin-6 (IL-6) (**C**) after PMX-DHP application

A 34-year-old Korean male with no underlying diseases was rescued from a fire in his home. When the firefighters arrived, he was lying in his bed unconscious and pulseless. Ventricular fibrillation was observed on an automated external defibrillator monitor, and 2 minutes of chest compression with 150 J shock was applied. Recovery of spontaneous circulation ensued just after defibrillation, and the patient was transferred to the emergency department (ED). Upon arrival, erythema with vesicles was found in his face, neck, chest wall, both axillary areas, and the back. In addition, he had a low estimated Glasgow Coma Scale score (3 points) and weak respiration, so intubation and mechanical ventilation support were applied. The initial blood pressure was 88/46 mmHg. Laboratory tests showed leukocytosis, elevated hepatic enzymes (aspartate transaminase 310 U/L, alanine transaminase 184 U/L) and relatively normal levels of C-reactive protein (0.07 mg/dL) and procalcitonin (0.07 ng/dL). On the other hand, the level of serum interleukin 6 (IL-6) was markedly increased to 1328 pg/mL. The initial arterial blood gas (ABG) showed hypoxemia with a low partial pressure of oxygen (PaO_2_ 37 mmHg) as well as metabolic acidosis with a serum lactate level of 12.6 mmol/L. The percentage of serum COHb was 59%, indicating severe CO poisoning. An initial chest X-ray showed diffuse bilateral infiltration, and chest computed tomography (CT) revealed diffuse ground-glass attenuation with an anterior–posterior density gradient by dense consolidation in dependent areas, which suggests an early state of ARDS (Figure 1). Therapeutic hypothermia was applied for prevention of hypoxic brain damage. Shock requiring infusion of norepinephrine exceeding the rate of 0.5 μg/kg/minute despite adequate fluid resuscitation was observed after sedation along with therapeutic hypothermia. Even with 100% of fraction of inspired oxygen (FiO_2_) and maximal positive end expiratory pressure, hypoxemia (PaO_2_ 61 mmHg) as well as hypercapnia (partial pressure of carbon dioxide 58 mmHg) progressed. Prone position ventilation and application of inhaled nitric oxide were considered, but were not implemented due to concern for rapid progression of shock. Therefore, we decided to conduct veno-veno extracorporeal membranous oxygenator (V-V ECMO) via both right and left femoral venous cannulation. Despite V-V ECMO application, hypoxemia and shock progressed. Thus, direct hemoperfusion with polymyxin B-immobilized fiber (PMX-DHP) was indicated and conducted immediately to prevent further development of cytokine storm. PMX-DHP (TORAYMYXIN^TM^ PMX-20R, TorayMedical, Tokyo, Japan) was applied with continuous renal replacement therapy (CRRT) via a nontunneled, double-lumen catheter inserted in left jugular vein, with a starting blood flow rate of 150 mL/minute for 24 hours. The patient was transferred to the intensive care unit (ICU) with ECMO and PMX-DHP. In the ICU on post-burn day 1, flexible bronchoscopy was done to assess degree of inhalation injury and to toilet the airway. Bronchoscopy revealed severe edema and congestion in the bronchial wall with carbon soot deposition. (Figure 2) With V-V ECMO maintenance and 24 hours of PMX-DHP, serum COHb percentage and IL-6 level normalized, and dramatic improvement on chest X-ray was seen within 96 hours. (Figure 3) On the seventh and eighth ICU days, the patient was weaned from V-V ECMO and the ventilator, respectively. On the 11th ICU day, the patient was moved to the general ward with a plan for systematic rehabilitation including respiratory rehabilitation. Upon transfer, the patient was alert without cognitive dysfunction, and electroencephalogram along with diffusion magnetic resonance imaging showed no definitive signs of hypoxic brain damage. However, both sensory impairment and motor weakness were observed in the right lower extremity with an abnormal result on electromyography suggesting right lumbar plexopathy. The patient was discharged from hospital with the plan of further rehabilitation through the outpatient clinic in the rehabilitation medicine department of hospital in other city.

## Discussion

Inhalation injury is a disastrous injury that occurs in one-third of all burn patients, and smoke inhalation injury contributes to increased mortality, up to 20 times higher than that of burn alone [5, 6]. Smoke inhalation injury can lead to not only long-term pulmonary dysfunction but also prompt respiratory complications such as pneumonia and acute respiratory distress syndrome. ARDS might be caused by contributing factors, including smoke toxicity, bronchorrhea, alveolar permeability, and inflammatory mediator expression. The incidence of ARDS in patients with burn and inhalation injuries who required mechanical ventilation was reported in the range of 34–43% [7]. In terms of ARDS, mortality is up to 40%; and intubation, fiberoptic bronchoscopy and mechanical ventilator support are currently used as initial treatments at the ED [8]. However, in cases of refractory, severe ARDS with maximal support of mechanical ventilation, ECMO should be considered as an additional option. Recently Dabras *et al*. suggested that ECMO is a viable therapeutic option and can contribute to improved survival rates in patients with ECMO for ARDS after inhalation injuries [4]. Cytokine storm, or cytokine storm syndrome, is caused by excessive circulating serum cytokines after inhalation injury and is also a significant complication. Progressive, widespread, systemic inflammation leads to low vascular permeability that is manifested as vasodilatory shock and progressive organ failure resulting in a poor prognosis [9]. PMX-DHP was originally developed to adsorb endotoxins released by Gram-negative bacteria during septic shock [10]. However, several studies recently reported that PMX-DHP might be useful for ARDS patients and for removal of cytokines [11–13]. Therefore, in patients with severe refractory ARDS and cytokine storm, early implication of PMX-DHP for removal of cytokines and improvement of oxygenation might be considered in addition to ECMO.

## Conclusion

We strongly suggest that early initiation of ECMO along with PMX-DHP even at the ED should be considered as a treatment option for severe ARDS with cytokine storm following inhalation injury to prevent mortality and to promote a better prognosis in burn patients.

## Data Availability

The datasets generated during and/or analyzed during the current study are available from the corresponding author on reasonable request.

## Abbreviations

CO: Carbon monoxide
ARDS: Acute respiratory distress syndrome
ED: Emergency department
ECMO: Extracorporeal membrane oxygenation
PMX-DHP: Direct hemoperfusion with polymyxin B-immobilized fiber
COHb: Carboxyhemoglobin
IL-6: Interleukin 6
ABG: Arterial blood gas
PaO2: Partial pressure of oxygen
CT: Computed tomography
V-V ECMO: Veno-veno extracorporeal membranous oxygenator
CRRT: Continuous renal replacement therapy
ICU: Intensive care unit
